# Microglial Dynamics and Role in the Healthy and Diseased Brain: A Paradigm of Functional Plasticity

**DOI:** 10.1177/1073858414530512

**Published:** 2015-04

**Authors:** Diego Gomez-Nicola, V. Hugh Perry

**Affiliations:** 1 Centre for Biological Sciences, University of Southampton, Southampton, UK

**Keywords:** microglia, proliferation, surveillance, synaptic pruning, neuromodulation, phagocytosis, neuroinflammation

## Abstract

The study of the dynamics and functions of microglia in the healthy and diseased brain is a matter of intense scientific activity. The application of new techniques and new experimental approaches has allowed the identification of novel microglial functions and the redefinition of classic ones. In this review, we propose the study of microglial functions, rather than their molecular profiles, to better understand and define the roles of these cells in the brain. We review current knowledge on the role of surveillant microglia, proliferating microglia, pruning/neuromodulatory microglia, phagocytic microglia, and inflammatory microglia and the molecular profiles that are associated with these functions. In the remodeling scenario of microglial biology, the analysis of microglial functional states will inform about the roles in health and disease and will guide us to a more precise understanding of the multifaceted roles of this never-resting cells.

## Introduction

**Figure 1.**
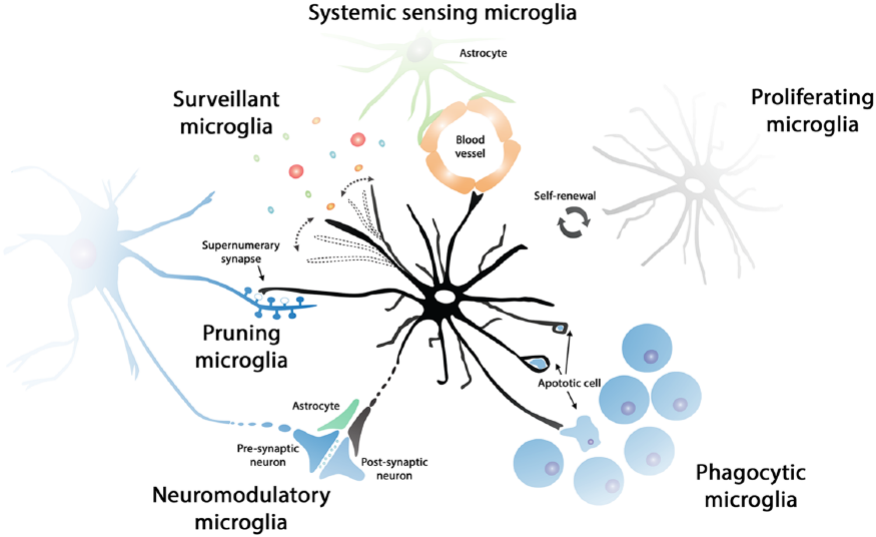
Functional states of microglia in the healthy brain. The population of microglial cells is maintained by self-renewal, without the contribution of bone-marrow-derived progenitors. Surveillant microglial cells constantly scan the brain microenvironment, in order to detect minor perturbations of CNS homeostasis. Surveillant microglia can, for example, detect the presence of neurotoxic substances or inflammatory mediators from the systemic circulation, being in close communication with the blood-brain barrier (systemic sensing microglia). Phagocytic microglia can detect and quickly remove damaged or dying neurons, preventing the damage to neighboring cells and helping maintain the high turnover of specific cell populations (i.e., neural precursor cells). The phagocytic capacity of microglia is particularly important in development (pruning microglia), when they can contribute to the removal of supernumerary synapses in certain neuronal pathways. Moreover, it has been suggested that microglia can have a direct or indirect modulatory role at the synapse, influencing neuronal activity (neuromodulatory microglia).

Microglia are the myeloid resident cell population of the central nervous system (CNS) parenchyma. The first identification of microglial cells was simultaneously reported by F. Robertson (Robertson 1900) and F. Nissl (Nissl 1899), who named them as “Staebchenzellen” based on the rod-like shape of their nuclei, describing them as reactive neuroglia. These cells later received the name of “microglia” from Pio del Rio-Hortega, a Spanish scientist from Santiago Ramon y Cajal’s school, differentiating them from the other glial cells and highlighting their potential to differentiate from ramified to amoeboid cells (del Rio Hortega 1932; del Rio Hortega and Penfield 1927; del Rio-Hortega 1920). Microglial cells are active sensors of the disturbances in their microenvironment, capable of elaborating a diverse spectrum of responses to restore tissue homeostasis (Hanisch and Kettenmann 2007; Kreutzberg 1996). In keeping with the long-lasting definition as “the macrophages of the brain” it is only recently that microglial cells have been shown to have many potentially important functions in the normal development, function, and repair of the CNS (Fig. 1). The understanding of microglial origin and functions in health and disease is experiencing a revolution, and many aspects of their physiology are being redefined. For example, the textbook dogma that microglial cells are of mesodermal origin, derived from hematopoietic stem cells in the bone marrow, has recently been rebutted by experiments demonstrating that microglia are mainly derived from the yolk sac, colonizing the neuroepithelium in early embryogenesis (Ginhoux and others 2010). Other ideas, like the use of the terms “resting” or “quiescent” to define the behavior of microglial cells in the healthy brain, are now obsolete, as it indicates a degree of inactivity that does not reflect the current in vivo observations showing that microglia use their motile processes to actively scan the microenvironment, and to interact with synapses and with oligodendrocyte-derived myelin (Davalos and others 2005; Fitzner and others 2011; Kettenmann and others 2013; Nimmerjahn and others 2005; Paolicelli and others 2011; Schafer and others 2012; Wake and others 2009). The remarkable potential of microglia to react to almost any form of disturbance of CNS homeostasis, infection, acute or chronic injury has often been viewed as an on-off switch, namely, “microglial activation” but this does not reflect the functional plasticity of these cells. The long-held assumption that microglial activation was detrimental and neurotoxic has dominated the scientific literature for many decades, stigmatizing the potential contribution of these cells to CNS physiology. In many experimental paradigms, the detrimental contribution of microglia has been demonstrated, as well as a clear neuroprotective function in others (Ransohoff and Perry 2009). The current literature has extensively reviewed this issue in the past, presenting microglial cells as “friend or foe” or as a “double-edged sword” trying to understand the determinants of the positive versus negative microglial contributions to brain pathology, with the goal of minimizing the harmful and favoring the beneficial (Crutcher and others 2006; Popovich and Longbrake 2008). However, capturing or promoting the beneficial effects alone is unlikely to be straightforward since the responses of microglia, like other tissue macrophages, are not linear, compartmentalized, or binary, but represent a highly plastic multifaceted response, finely tuned by the nature of the stimulus, the molecular repertoire that is engaged, and the prior state of the cell (Gordon 2003; Ransohoff and Perry 2009). This complex nature becomes particularly difficult to understand when taking into account the status of the immune-privilege of the brain that defines and tightly controls innate and acquired immune responses, but also their responses to the influence of pathological processes from peripheral organs (Perry and Teeling 2013).

We suggest that it will be useful to describe the activity of microglial cells in diverse conditions, reacting to diverse stimuli, as functional states defined by specific functions, rather than limited molecular profiles or morphological criteria (Fig. 1). In this review, we provide support to this approach by describing currently data available from the recent investigation of microglial functions in the CNS, assuming that the list of microglial functions will increase in diversity and complexity in the following years.

## Microglial Functional States in the Healthy and Diseased Brain

### Where Do Microglial Cells Come From? Migrating and Proliferating Microglia

The population of microglial cells in the adult murine CNS accounts for 5% to 12% of the total number of glial cells, depending on the region analyzed (Lawson and others 1990). In humans, the microglial population accounts for 0.5% to 16.6% of the total of cells in the brain, showing similar regional variability as that of rodents (Mittelbronn and others 2001). Microglia in the human brain show higher densities in the white matter than in the grey matter, in contrast to rodents (Mittelbronn and others 2001). But, where do these cells come from and how they maintain the resident microglial population? Although a clear answer to the origin of microglia is getting much closer some questions remain.

Starting from pioneering studies from Del Rio-Hortega, it was assumed for decades that the adult microglial population originated from embryonic and perinatal waves of hematopoiesis and infiltration of circulating blood monocytes, followed by microglial differentiation (for review, see Ginhoux and others 2013). Macrophage-like cells were reported in the brain parenchyma from early development (E12-16), as F4/80^+^ cells with amoeboid morphology (Morris and others 1991; Perry and others 1985; Wang and others 1996). Interestingly, several studies reported the presence of amoeboid cells expressing macrophage/microglia markers in the primitive developing brain (E8.5/E9.0), suggesting the idea of an early development and a yolk sac origin of the resident microglial population (Alliot and others 1999; Chan and others 2007; Mizutani and others 2012). The definitive evidence supporting the yolk sac as origin of microglial cells was reported by Ginhoux and coworkers, and also highlighted the dependence of this developmental system on blood circulation, which is not required for the establishment of other tissue macrophage populations (Ginhoux and others 2010). Moreover, the idea that microglia and other tissue macrophages constitute independent cell lineages is supported by recent studies highlighting the differences between macrophages derived from yolk sac and definitive hematopoiesis, the latter being dependent on the transcription factor Myb (Schulz and others 2012).

The microglial population acquires its definitive composition, in terms of numbers and phenotype, soon after birth. A wave of microglial proliferation has been reported at early postnatal stages, but it is unclear if this could account for the increase in numbers of microglial cells, or rather suggesting the possible contribution of blood-derived monocytes (Alliot and others 1999; Tambuyzer and others 2009). Although the postnatal infiltration of circulating monocytes and further differentiation to microglia has been described under certain experimental conditions, populating the corpus callosum (Ling and others 1980), or repopulating the whole microglial population (Beers and others 2006), further quantitative data are missing in the literature, a key to understand the final composition of the microglial population. Recent experiments using transgenic tagging of E7.25- versus E8.5-derived cells provided robust support to the idea that the microglial population is derived almost exclusively from the yolk sac, excluding the contribution of blood-derived monocytes (Ginhoux and others 2010; Kierdorf and others 2013; Schulz and others 2012). The selective nature of the blood-brain barrier (BBB) could support this exclusive behavior, as complementary studies have defined that the rodent BBB is established at E13.5, before the release of monocytes into circulation and after the invasion of yolk sac-derived cells (Daneman and others 2010).

The picture of the origin and maintenance of the human microglial population is not as clear as in rodents. Microglial cells have been described in the brain from the 3rd gestational week, appearing in the spinal cord at the 9th week, completing the colonization of the embryonic CNS at around 22 weeks (Hutchins and others 1990; Rezaie and Male 1999). Microglial cells with ramified morphology can be only observed closer to term, around the 35th week (Esiri and others 1991). Although these studies suggest very early waves of colonization of the CNS by yolk sac–derived hematopoietic precursors, preceding the onset of bone-marrow hematopoiesis, and are in accord with data arising from rodents, the exact dynamics of the human adult microglial population is still to be elucidated.

**Figure 2.**
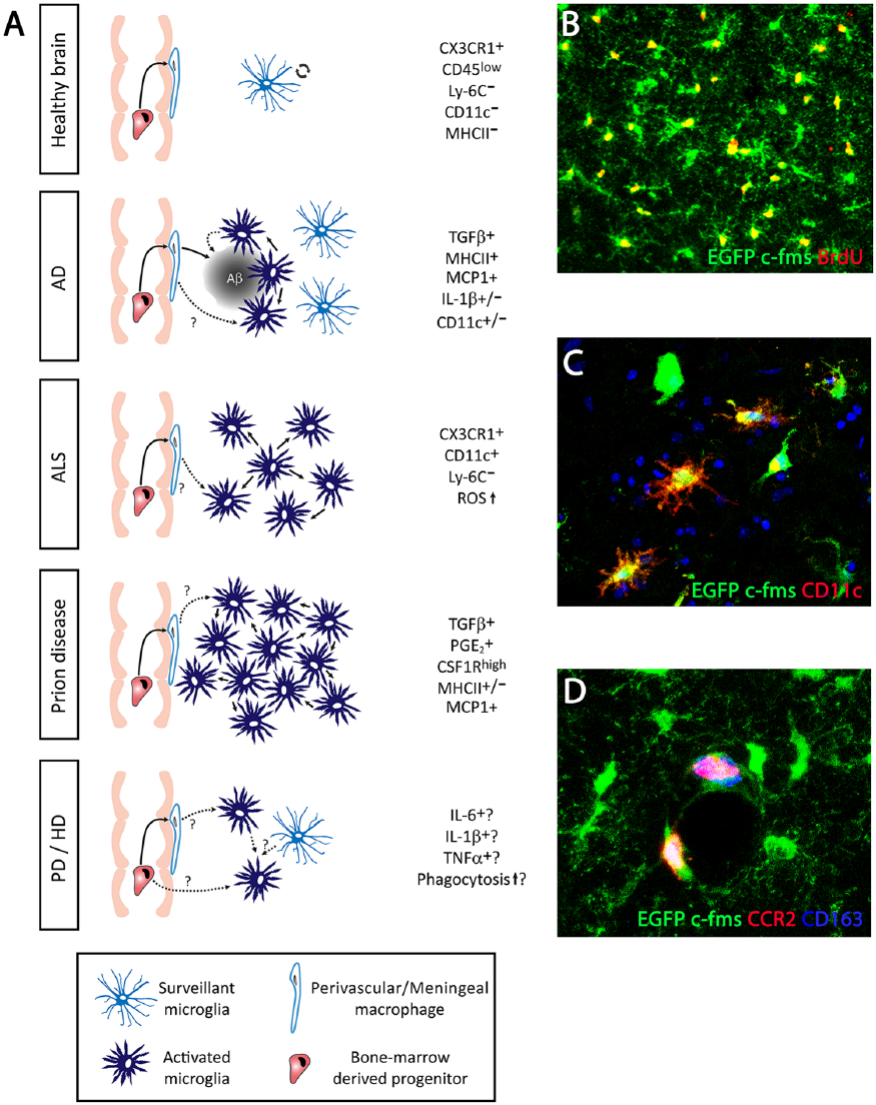
Dynamics and functions of microglia in chronic neurodegenerative diseases. (**A**) In the normal brain, the microglial population has a surveillant phenotype, maintaining homeostasis. The microglia population is maintained by self-renewal, while the perivascular macrophages can be renewed by bone-marrow-derived progenitors. In Alzheimer’s disease (AD) microglia proliferate and accumulate around plaques of amyloid β (Aβ), participating in the attempted removal of the misfolded protein. Perivascular macrophages have a more efficient phagocytic activity than microglial cells in AD. In AD, the microglial population is increased without a contribution from bone-marrow-derived cells. Microglia are expanded and activated during the course of amyotrophic lateral sclerosis (ALS), without a contribution from circulating progenitors. In prion disease, the microglial population is expanded dramatically by local proliferation (**B**; BrdU^+^), being primed to give an exaggerated response to systemic inflammatory events. Little evidence is available about the expansion/renewal of the microglial population during Parkinson’s or Huntington’s disease, or the dominant inflammatory phenotype. In general, the microglial population does not generate a uniform response and a diverse inflammatory prolife can coexist during disease (**C**; CD11c^+^ vs. CD11c^−^ microglia). For all the neurodegenerative diseases considered, little evidence is available about the possible contribution of perivascular macrophages (**D**; CD163^+^ CCR2^+^) to the expansion/renewal of the microglial population (**D**; CD163^−^ CCR2^−^), although both populations have different activation and proliferation patterns. (**B**-**D**) Representative examples evidenced in prion disease, detecting microglial cells by the transgenic expression of EGFP under the c-fms promoter.

The recent redefinition of the microglial origin suggests an idea: the microglia population must be maintained by self-renewal of proliferating resident cells (Figs. 1 and 2). The relative contribution of bone-marrow-derived cells (BMCs) to the pool of perivascular macrophages (PVMs), meningeal macrophages (MMs), or parenchymal microglia in health and disease is a matter of intense debate (Fig. 2). As discussed above, microglia originate from the yolk-sac and function largely independently of BMCs in the healthy brain (Ginhoux and others 2010), pointing to in situ microglial proliferation as the mechanism regulating the population turnover, with little or no contribution from circulating progenitors (Lawson and others 1992; Prinz and Mildner 2011). However, limited evidence is available in the literature defining the exact rates and regulation of microglial turnover in the healthy brain. Seminal work from Lawson and others (Lawson and others 1992), using H^3^ thymidine combined with immunohistochemistry for F4/80, demonstrated that microglia proliferate in the healthy brain, but more slowly that other tissue macrophages: 0.05% of the microglia is proliferating at a given time, 20 times less than the lowest labeling index for any other resident macrophage population studied. However, these figures need to be reevaluated using more reliable detections methods to avoid underestimating the numbers of proliferating microglia. Moreover, we do not have precise information about the mechanisms/pathways regulating microglial proliferation in the steady state, or its counterpart microglial apoptosis, necessary to maintain a balanced population. Undoubtedly, the knowledge about microglial origin has expanded dramatically in the last years but there is still much to be done to reach a full understanding of microglial dynamics.

The understanding of the microglial population dynamics is yet more complex when studying the diseased brain. Recent studies support the idea that there is a minor or even absent contribution of BMCs to the microglial population in mouse models of Alzheimer’s disease (AD) (Mildner and others 2011), motor neuron disease and axotomy (Ajami and others 2007), prion disease (Gomez-Nicola and others 2013, 2014), and stroke (Li and others 2013) (Fig. 2). In other diseases, like multiple sclerosis (MS), the evidence using genetic labeling of the different populations shows that expansion of the microglia/macrophage population is the result of a combination of both microglial proliferation and infiltration of circulating monocytes (Ajami and others 2011).

A useful example to illustrate the shift in thinking about microglial cell dynamics is in the study of AD. Although local proliferation was assumed to be responsible for the consistently documented expansion of the microglial population observed in AD, mainly accumulating around plaques (Bolmont and others 2008; Frautschy and others 1998), direct evidence of proliferating microglial cells in animal models (Kamphuis and others 2012) or human postmortem samples (Gomez-Nicola and others 2013) of AD was reported only recently. The detection of proliferating microglia in AD correlates with the up-regulation of the transcription factor PU.1 and the mitogens colony-stimulating-factor-1 (CSF1) and interleukin-34 (IL-34), key components of the pathway regulating microglial proliferation (Gomez-Nicola and others 2013). Another determinant of microglial proliferation, colony-stimulating factor 1 receptor (CSF1R), has also been found to be up-regulated in microglial cells during AD, indicating a prominent activity of this pathway (Akiyama and others 1994). These findings compare well with evidence reported in experimental models of prion disease, a paradigmatic chronic neurodegenerative disease that progress with a 10-fold expansion of the microglial population. Microglial proliferation in prion disease is maintained by the activity of the CSF1R signaling pathway, and specific antagonism of the receptor, using either blocking antibodies or the selective CSF1R inhibitor GW2580, highlights the detrimental contribution of microglial cells to the disease (Gomez-Nicola and others 2013). A reduction in the numbers of proliferating microglia, by specifically inhibiting CSF1R, delayed the onset of behavioral deficits and extended the time to terminal disease. The reported similar activity of the CSF1R pathway in experimental models of AD and prion disease, human prion disease (variant Creutzfeldt-Jakob disease; vCJD) and human AD, suggests common pathways controlling microglial proliferation and activation in chronic neurodegeneration (Gomez-Nicola and others 2013) (Fig. 2). Furthermore, circulating progenitors do not contribute to the microglial population in prion disease, while they define the expansion of the PVMs population (Gomez-Nicola and others 2014). These studies pinpoint the importance of the control of microglial proliferation during neurodegeneration, offering new avenues for the regulation of the innate immune response in the brain. The analysis of the experimental models of prion disease offers an attractive perspective for the future, as they exhibit the main pathological features observed in many human neurodegenerative conditions (prion disease, AD, Parkinson’s disease [PD]): protein misfolding, synaptic dysfunction, neurodegeneration, and an innate inflammatory reaction (Ransohoff and Perry 2009).

Targeting the expansion of the microglial population has been used as an experimental tool to dissect the contribution of microglial cells to brain disease (Fig. 2). For example, a repopulation method with SOD-1-expressing BMCs in microglia-devoid PU.1^−/−^ mice was used to define a detrimental contribution of microglia to the progression of experimental amyloid lateral sclerosis (ALS) (Beers and others 2006). Approaches in which microglial proliferation has been blocked, either by the transgenic expression of thymidine kinase (TK) and “suicide” of proliferating CD11b^+^ cells (Gowing and others 2008; Grathwohl and others 2009), or by the administration of the nonspecific blocker of mitosis Ara-C (Audet and others 2012), indicated a neutral or beneficial role of microglia in AD or ALS. However, the methods used in these studies did not take into account the inherent technical limitations. First, the use of CD11b-TK mice leads to a massive and uncontrolled death of microglia in the context of a CNS with ongoing neurodegeneration (Gowing and others 2008; Grathwohl and others 2009): this is not a “physiologically silent” way to address the contribution of microglial cells. Additionally, the activation of the TK transgene in CD11b-TK mice is achieved by administration of ganciclovir: this agent was recently identified to have a potent antiproliferative impact on microglia during brain pathology (Ding and others 2014). Second, the use of Ara-C causes a shift in the activation phenotype of microglia toward a detrimental pro-inflammatory profile, independent from its effects on cell proliferation (Gomez-Nicola and others 2013), probably explaining its detrimental effects on a model of ALS (Audet and others 2012). Other alternative approaches have studied the impact of increasing the proliferative activity of microglia with recombinant CSF1. These studies also suggest a detrimental role for microglia in the pathophysiology of ALS (Gowing and others 2009), although these experiments also affected the contribution from CSF1-responsive peripheral cells. As previously suggested, the use of experimental strategies targeting the pathway(s) regulating microglial proliferation would provide a clearer readout of the overall contribution of microglia to the pathogenesis of diverse neurodegenerative conditions.

In a number of important neurodegenerative conditions the study of microglial population dynamics remains mostly unexplored. For example, PD is characterized by the presence of morphologically activated microglia (Fig. 2), evidenced in human postmortem samples (McGeer and others 1988), and by in vivo PET imaging, showing increased binding of the microglial-specific ligand PK-11195 without any correlation with the clinical symptoms (Gerhard and others 2006). Also, in Huntington’s disease (HD), progressive morphological activation of microglia and increase in their number has been evidenced in human brain from early presymptomatic stages of the disease, suggestive of a causative role for these cells in the pathology (Sapp and others 2001; Tai and others 2007). Binding studies of PK11195 to microglia using PET imaging in HD patients suggests that microglial activation correlates with the severity of the disease (Pavese and others 2006), leading to the suggestion that they might provide a useful diagnostic tool to predict disease onset (Politis and others 2011). However, the contribution of BMCs infiltration versus microglia proliferation to the expansion of the PVMs, MMs, or parenchymal microglial population in PD or HD remains unexplored and is of importance to fully understand the innate immune response in these brain pathologies (Fig. 2). Analyzing PVMs, MMs, and microglial proliferation under pathological conditions with widespread degeneration is critical for understanding how innate inflammation contributes to the onset and progression of the disease. Recent studies have highlighted the ability of PVMs to clear amyloid β (Aβ) in experimental models of AD (Mildner and others 2011) and show the need for a better understanding of the differential contribution of BMCs, MMs, and PVMs for the expansion of the microglial population and providing a key link with systemic inflammatory events.

### Surveillant Microglia

The cytoarchitecture of the microglial population is regularly organized, forming a tightly controlled mosaic, independent of cell layers or blood vessels (Lawson and others 1990). However, the microglial population shows remarkable anatomical diversity. Microglial cell density can vary across regions, representing a 12% of total cells in the substantia nigra or 5% of the corpus callosum of rodent brains (Lawson and others 1990) or 0.3% of the total cells in the cerebellar grey matter and 16.9% in the medulla oblongata of human brains (Mittelbronn and others 2001). Microglial morphology is also diverse, with elongated and orientated cells in the white matter or amoeboid cells in the circumventricular organs, in contrast to the more abundant radially orientated arborized morphology (Lawson and others 1990).

The application of live imaging techniques to the study of the brain provided dynamic details of the microglial population. In both mice and zebrafish, microglia constantly and rapidly scan the microenvironment with their processes, while keeping the soma in a fixed position (Davalos and others 2005; Nimmerjahn and others 2005; Wake and others 2009) (Fig. 1). Contact between processes is avoided during the scanning of the parenchyma, maintaining the mosaic distribution and the cell size (Nimmerjahn and others 2005). However, ageing has been shown to affect microglial stability, leading to a disruption of the mosaic organization, a decrease in the motility of microglial processes, and a remarkable increase in the motility of their somas (Hefendehl and others 2014). Although the dynamic process of microglial surveillance of the brain parenchyma has been characterized in detail, we have less information about the elevated energy expenditure associated with actin polymerization (Hines and others 2009) and about the microglial mechanisms to control this inherently high metabolic rate.

The maintenance of the surveillant microglial phenotype is achieved by diverse soluble or membrane-bound factors with neuronal or non-neuronal origin (Hanisch and Kettenmann 2007; Kettenmann and others 2011). However, the regional heterogeneity of the brain (different neurotransmitter environment, myelin content, BBB properties, etc.) and the impact of systemic events on the microglial receptor signature must be translated into region and time-specific mechanisms of surveillance, which are not fully understood to date. Microglial dynamics can be stimulated by factors like ATP and other nucleotides (Davalos and others 2005; Fontainhas and others 2011) or reduced by factors including CX3CL1 (Liang and others 2009), with other well-know modulatory systems like CD200, CD47, or GABA still not fully understood (Kettenmann and others 2011).

### Pruning and Neuromodulatory Microglia

The initial phases of postnatal brain development are characterized by a process of remarkable plasticity involving neuronal and glial cell death, and synaptic pruning or remodeling. In an activity-dependent manner, extranumerary synapses are eliminated, while the remaining ones are strengthened to form the adult connectivity (Hua and Smith 2004; Katz and Shatz 1996). The mechanism regulating the removal of synapses was elusive, but now microglial cells have been proposed as key executive players. The association of microglia with areas subjected to intense postnatal cell death and synaptic remodeling was evidenced for many years (Dalmau and others 1998; Fiske and Brunjes 2000; Perry and others 1985). The involvement of microglia in the active elimination of extranumerary synapses was proposed in the context of the study of the developing retinogeniculate system, where a complement-dependent remodeling process leads to eye-specific segregation in the dorsal lateral geniculate nucleus (Stevens and others 2007) (Fig. 1). In this system, microglia were later found to engulf some synapses in a complement (C3)-dependent manner, directly shaping neuronal connectivity (Schafer and others 2012). Additionally, the CX3CR1-CX3CL1 system has also been suggested to regulate synapse pruning by microglia, as deficiencies in CX3CR1 led to a reduction of microglial density, which is in turn associated with a modest and transient defect in developmental synaptic connectivity (Paolicelli and others 2011). More recently, the CX3CR1-CX3CL1 system has been directly evidenced to determine functional brain connectivity and behavioral changes (Zhan and others 2014). In addition to microglial pruning activity in the retinogeniculate system, astrocytes were recently found to also remove synapses, involving the MEGF10 and MERTK phagocytic pathways (Chung and others 2013). Although synaptic pruning seems associated with development of CNS circuitry, evidence at the ultrastructural level suggests a role of microglia in the reorganization of adult circuits following sensory loss (Tremblay and others 2012) or during ischemia (Wake and others 2009).

The precise role of microglia in the process known as “synaptic stripping”, the separation of the presynaptic terminal from an injured postsynaptic neuron, is a matter of controversy (Perry and O’Connor 2010). There are important species differences in mouse and rat and the involvement of astrocytes has been overlooked in some studies (Yamada and others 2011). The initial definition of synaptic stripping by microglial processes in the injured facial nerve (Blinzinger and Kreutzberg 1968) was supported by findings in MeCP2-deficient mice, evidencing the impact of microglial-derived glutamate on the synaptic element (Maezawa and Jin 2010). Further evidence from live imaging supported a transient and rapid interaction or contact of microglial processes with axon terminals and dendritic spines, being modulated by neuronal activity (Wake and others 2009). In the disease context, the interaction of microglial processes with axon terminals has been shown altered in a model of ischemia, suggesting an active role of this interaction in the preservation of the synaptic connectivity (Wake and others 2009). However, in a prion disease model of chronic neurodegeneration, in which extensive synaptic degeneration occurs prior to death of the neuronal soma, the synapses degenerate and are enveloped by the spine postsynaptic density without the involvement of microglial cells (Siskova and others 2009).

Although the microglial pruning has been described during postnatal development, it is still unclear how widely this mechanism is involved in earlier stages of development, during disease, and in pathologies with a component of synaptic degeneration. Without doubt this arena will attract significant attention in the next years and will provide valuable information to fully understand the link of the inflammatory response with neuronal degeneration.

Further to the pruning role of microglia, some evidence suggests a direct participation in the regulation of neuronal activity, in a quad-partite model together with astrocytes (Bechade and others 2013; Schafer and others 2013) (Fig. 1). Microglial activation with LPS causes an increased production of ATP that signals to astrocytes to produce an increased excitatory postsynaptic current in hippocampal neurons (Pascual and others 2012). This direct and intimate relationship with astrocytes and neurons is supported at the ultrastructural level, where microglial processes have been shown in contact with excitatory synaptic elements (Tremblay and others 2010). However, other electron microscopy studies show that only a small percentage, 3.5%, of synapses receive direct contact by a microglial process, questioning the overall impact or direct relevance of microglia on synaptic activity (Sogn and others 2013).

### Phagocytic Microglia

The phagocytic activity of microglia is one of the features in common with their cellular cousin, the macrophage, and helps eliminate bacteria during infections, necrotic and apoptotic cells during development or disease (Sierra and others 2013). Phagocytic microglia removes apoptotic debris in the developing and adult brain (Fig. 1), keeping cell death silent and avoiding the deleterious secretion of pro-inflammatory cytokines and chemoatractants driving the migration of T cells; therefore, phagocytosis has a net beneficial effect (Chan and others 2006; Magnus and others 2001). However, data arising firstly from in vitro studies (Neher and others 2011) and more recently from in vivo experiments (Fricker and others 2012; Neher and others 2013) support the notion that microglia can actively remove endangered but potentially viable neurons, contributing to brain pathologies with a neuroinflammatory component. These ideas lead to the use of the term “phagoptosis” to define the selective attack and removal of damaged but viable neurons by microglia, resembling similar cell-cell interactions observed in peripheral organs (Brown and Neher 2012).

The study of the role of phagocytic microglia in the healthy brain is perhaps exemplified by the study of the hippocampal neurogenic niche (Sierra and others 2013) (Fig. 1). The neurogenic cascade at the hippocampal subgranular layer (SGL) leads to the generation of a population of early neural precursor cells (NPCs), which become finely selected by apoptosis, before completing the maturation to granule cells (Kempermann and others 2004; Ming and Song 2011). In this niche, ramified phagocytic microglia rapidly and efficiently remove dying NPCs in a non-inflammatory fashion (Sierra and others 2010). When challenged with LPS, NPCs undergo increased apoptosis (Sierra and others 2010), although it is unclear if as a consequence of the indirect production of pro-inflammatory cytokines or direct phagoptosis.

In addition to the direct removal of dead/damaged/alive cells, phagocytic microglia can engulf and prune synapses (Schafer and others 2012), clear axonal and myelin debris (Hosmane and others 2012; Nielsen and others 2009) or clear potentially toxic proteins such as amyloid beta (Aβ) (Sierra and others 2013). In the case of AD, the plaque burden increases with age, in both mouse models and human patients, indicating the rather ineffective phagocytic activity of microglia. In other models of chronic neurodegeneration, like the prion disease model, microglia have limited abilities to remove misfolded prion protein (PrP^sc^) (Hughes and others 2010). Aβ deposits have been shown to have a potent chemoattractant activity on microglia, although their removal by phagocytosis has not been clearly evidenced in vivo (Sierra and others 2013). However, the removal of Aβ can be improved by further challenge of microglia with high doses of LPS (Herber and others 2004) or the induction of IL-1β (Shaftel and others 2007). Although a significant body of literature suggested that BMCs can play a leading role in the removal of Aβ, therefore complementing the poor phagocytic activity of microglia (Simard and others 2006; Simard and Rivest 2006), more recent evidence from Prinz and colleagues support a differential contribution of perivascular macrophages and parenchymal microglia, not BMCs, to the clearance of Aβ (Mildner and others 2011). In fact, the regulation of the phagocytic activity of microglia appears as a key genetic determinant of AD pathology. Recent studies link genetic variants of TREM2, a protein regulating the activation and phagocytic functions of myeloid cells, with the risk of developing AD (Guerreiro and others 2013; Jonsson and others 2013). TREM2 has a balancing role between phagocytic and pro-inflammatory microglial activities and is expressed in microglia around plaques (Frank and others 2008). Similarly, dysregulation of the complement system in humans has been associated with AD (Lambert and others 2009; McGeer and McGeer 2002). However, as discussed above, no clear consensus defines the overall role of microglial phagocytosis in the diseased brain.

These conflicting findings, supporting a beneficial or a detrimental contribution of phagocytic microglia, need to be investigated and validated in detail in diverse in vivo experimental paradigms, escaping from confounding in vitro systems, before considering the application of anti-phagocytosis neuroprotective therapies, for example. Moreover, the use of refined experimental approaches to directly study microglial phagocytosis (Sierra and others 2010), rather than studying immunological markers such as CD68 (with still ill-defined functions in microglia), will shed light on the understanding of phagocytic microglia in health and disease.

### Inflammatory Microglia

Although the above-described functional states of microglia are significant contributions to our understanding the physiology of these cells, it is the inflammatory functions of microglia that dominate the scientific literature (Kettenmann and others 2011; Ransohoff and Perry 2009). In this section, we aim at providing some ideas, comparing the microglial inflammatory reaction in different pathologies without providing a comprehensive review of all the possible brain pathological states, which can be found in the literature (Hanisch and Kettenmann 2007; Kettenmann and others 2011; Ransohoff and Perry 2009). But, before reviewing the inflammatory functions of microglia during brain pathology, it may be informative to pay attention to the inflammatory profile of microglial cells in the healthy brain. Recent analysis of the microglial transcriptome show a profile dominated by RNAs encoding proteins for sensing endogenous ligands and microbes collectively referred as the “microglial sensome” (Hickman and others 2013). During ageing, microglia up-regulate the expression of microbe-recognition genes, together with genes involved in neuroprotection (Hickman and others 2013). A detailed comparison of the transcriptomic profile of microglia has also recently provided evidence about a unique functional signature in microglia, dominated by the activity of TGF-β (Butovsky and others 2014). This microglial gene signature, common to murine and human cells, allows the specific differentiation of microglia, when compared with other myeloid or immune cells, resident brain cells (oligodendrocytes, astrocytes, and neurons), microglial cells lines, or recruited monocytes, highlighting the particularities of the microglial population (Butovsky and others 2014). Compared to other tissue macrophages, microglia in the healthy brain have a down-regulated expression of molecules like CD45, Fc receptors, or MHC class II (Perry and Teeling 2013). The interaction of astrocytes and neurons with microglia provides a regulatory system to maintain the inflammatory pathways of microglia under control. Molecules like NGF or BNDF inhibit the expression of MHCII and its co-stimulatory molecules B7 and CD40 in microglia, respectively (Neumann and others 1998; Wei and Jonakait 1999). Also, neurotransmitters like GABA can control the inflammatory functions of microglia (Pocock and Kettenmann 2007). Additionally, the role of cell–cell interactions in the control of the microglial inflammatory phenotype has been studied in depth. For example, neuronal signaling using the CD200-CD200R or Siglecs systems inhibits the inflammatory activation of microglia, through the use of ITIM motifs (immunoreceptor tyrosine-based inhibitory motif) (Billadeau and Leibson 2002). When the control of these systems is lost, during neuronal degeneration for example, the inhibitory control is released from microglia, unleashing an inflammatory reaction (Bhaskar and others 2010; Zhang and others 2011). However, the idea that the microglial cell needs to be restrained is probably driven by the initial views of these cells as “the bad guys” and a more detailed reevaluation of these regulatory systems, together with novel ones, will inform about the maintenance of the brain homeostasis.

**Figure 3.**
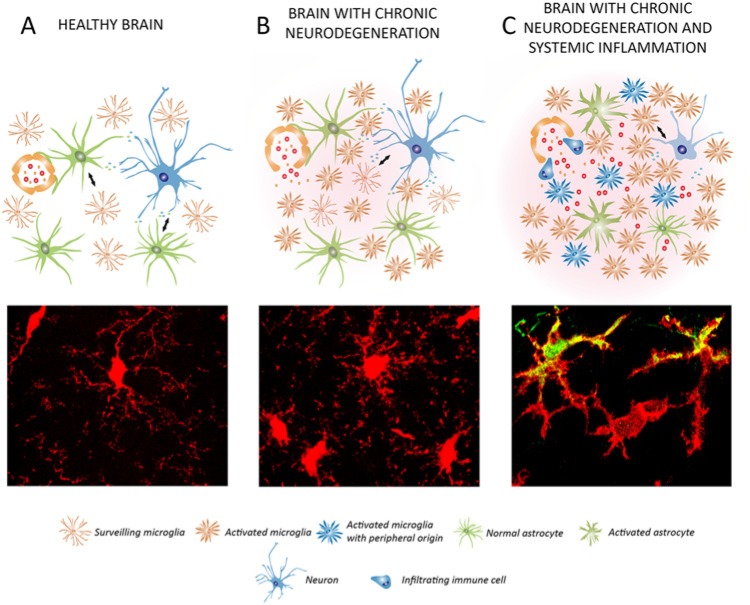
Impact of systemic inflammation on the progression of chronic neurodegeneration: microglial priming. Schematic representation of the cross-talk of microglial cells with neurons and astrocytes in the healthy brain (**A**), during chronic neurodegeneration (**B**), and when chronic neurodegeneration is combined with a systemic inflammatory event (**C**). (**A**) In the healthy brain, surveillant microglia maintain the brain homeostasis and are renewed by local proliferation. Astrocytes and microglia communicate with neurons to support their function and survival, among other functions (see Fig. 1). (**B**) In chronic neurodegeneration, microglia activate an inflammatory program and become primed. The microglial population is expanded mainly by local proliferation. Astrocytes lose control of the blood-brain barrier and inflammatory mediators and cells enter into the brain. Neurons undergo a progressive but limited damage. (**C**) When a systemic inflammatory event is combined with chronic neurodegeneration, primed microglia are further activated and damage endangered neurons, accelerating the pathology. The microglial population can be supplemented by bone-marrow-derived cells. Astrocytes become activated and further contribute to neuronal damage. (**A**-**C**) Microglial cells (red) exemplifying the different conditions are shown at the bottom. In **C**, primed microglial cells are shifted to a pro-inflammatory phenotype, expressing IL1β (green). The legend for the different cell types and phenotypes is provided at the bottom.

The inflammatory functions of microglia have special relevance for the understanding of the progression of neurodegenerative diseases. Despite a long-standing interest in the inflammatory response in AD, and the extensive research focused on understanding the role of microglia in this disease, the scientific community has failed to shed clear and uniform light into their contribution to the disease (Akiyama and others 2000; Heneka and O’Banion 2007; Ransohoff and Perry 2009). The neuropathology of AD shows a robust innate immune response characterized by the presence of activated microglia, with increased or de novo expression of diverse macrophage antigens (Akiyama and others 2000; Edison and others 2008), and at least in some cases production of inflammatory cytokines (Dickson and others 1993; Fernandez-Botran and others 2011). The fact that NSAIDs (nonsteroidal anti-inflammatory drugs) protect from the onset or progression of AD (Hoozemans and others 2011) suggests that inflammation is a causal component of the disease rather than simply a consequence of the neurodegeneration. The recent demonstration of several innate immune genes in genome-wide association studies (GWAS) of AD also implicates inflammation as causal in the disease (Perry and Holmes 2014). Additionally, a growing body of evidence suggests that systemic inflammation may interact with the innate immune response in the brain to act as a “driver” of disease progression and exacerbate symptoms (Holmes and others 2009; Holmes and others 2011) (Fig. 3). Studies in animal models show evidence of interactions between systemic inflammation and inflammation in the brain and importantly provide biologically plausible mechanisms for its contribution to the progression of neurodegeneration (Perry and Teeling 2013). The impact of systemic inflammation means that any neuropathology studies on the inflammatory response in the AD brain must take into account systemic co-morbidities that may influence the microglia phenotype (Fig. 3).

The definition of the brain inflammatory profile in AD shows conflicting ideas in the literature, probably arising from the heterogeneity of the postmortem samples and the difficult application of the detection methods (Boche and others 2013). Some authors have associated AD with a pro-inflammatory phenotype, characterized by expression of IL-1β and complement proteins, with a direct association with Aβ plaques in human samples (Griffin and others 1989; Griffin and others 1995; McGeer and others 1989). By contrast, other groups have reported the up-regulation of genes linked to an anti-inflammatory phenotype, arginase 1 or the transforming growth factor-β (TGF-β), in association with AD (Colton and others 2006; Wang and others 2003). The study of transgenic models of amyloidosis, modeling some aspects of AD, shows morphological activation of microglia that reproduce the deposition of Aβ (Jucker 2010; LaFerla and Oddo 2005; Perry and others 2007). However, the associated cytokine profile is by no means clear, as in the human AD brain the inflammatory response cannot be classified as strictly M1-like or M2-like (Sudduth and others 2013), with the changes in expression level compounded by the various detection methods (for review about research methods to study microglial biology, see Ransohoff and Perry 2009).

Although the precise inflammatory phenotype of microglia in AD seems elusive, the link of AD with inflammation seems clear, as highlighted by a recent study on the gene signature of ageing and AD, using microarray technology (Cribbs and others 2012). These results support the notion of an activation of the innate inflammatory response in microglia as a prelude to the subsequent development of AD (Cribbs and others 2012). Furthermore, studies on incipient AD (iAD) postmortem samples show a strong correlation between genes associated with the microglial response and the progression into AD (Blalock and others 2004). The concept of the interconnection of AD and the innate immune response is further supported by evidence from GWAS implicating genes involved in innate immunity (Lambert and others 2009). These promising studies are opening new avenues into the understanding of the impact of the innate immune response in AD, while supporting the need for future exploration.

In PD, some studies suggest that microglia have a pro-inflammatory phenotype, which is potentially driving neuronal injury (Hunot and others 1996; Mogi and others 1994), although no mechanistic study has yet addressed microglial contribution to the disease progression in humans. The interpretation of the inflammatory response in PD is complicated by the fact that PD has a late onset and that most studies analyzed end-stage samples, representing a brain that has been suffering from the disease for many years. Ageing alone has an impact on the phenotype of microglia, and systemic comorbidities, which can influence the microglial physiology, have not been taken into account in the previous studies focusing on PD (Perry 2012). The clinical course of PD is often associated with other comorbidities, like chronic constipation or aspiration pneumonia, driving a peripheral inflammatory response that might impact the brain microglial responses and the progression of PD (Perry 2012) (Fig. 3).

The understanding of the role of microglia in PD comes from the study of experimental animal models, although they fail to accurately reflect the neuropathology of PD as described in humans. PD is characterized by a slowly evolving degeneration of the substantia nigra (SN) dopaminergic neurons, an aspect not replicated in these models using either neurotoxic toxins or inflammatory challenges. The use of intracerebral neurotoxins, most commonly 6-hydroxydopamine (6OHDA), 1-methyl-4-phenyl-1,2,3,6-tetrahydropyridine (MPTP), or rotenone, provides a rapid degeneration (within a few days) of the SN dopaminergic neurons. Microglial activation has been described in the 6OHDA and MPTP models of PD (McGeer and others 2003; Walsh and others 2011), although limited information is available regarding the inflammatory phenotype of these cells, in contrast to their morphological features, which have been described in detail. Studies modulating microglial activity with minocycline, an antibiotic having anti-inflammatory actions, provided contrasting, model-dependent results, about the contribution of innate inflammation to the acute neurodegeneration of dopaminergic neurons in the SN (Sriram and others 2006; Wu and others 2002). Systemic inflammation, induced by administering IL-1β, was shown to impact the survival of dopaminergic neurons in the 6OHDA model, providing clear evidence of the influence of immune-to-brain communication on the progression of PD (Pott Godoy and others 2008). Additionally, the generation of transgenic mouse models of PD, based on the identification of genes linked with familial PD, also provides a promising approach to model chronic neurodegeneration (Dawson and others 2010). For example, transgenic overexpression of α-synuclein, a protein linked genetically to PD, leads to microglial activation and production of TNF-α in the SN (Su and others 2008), although little neuronal death is observed. Both transgenic and inflammatory models of PD can capture aspects of the disease, but fail to provide a comprehensive picture in which to address the roles of the innate immune response, in the context of a slowly evolving neurodegenerative condition. To summarize, the contribution of microglial cells to the onset or progression of PD is not yet established. Further research into the effect of systemic comorbidities (Fig. 3) and in refining the experimental animal models will help understand the roles of the innate immune response in PD.

In HD, a progressive morphological activation of microglia and increase in their number has been evidenced in the brain from early pre-symptomatic stages, suggestive of a causative role for these cells in the disease (Sapp and others 2001; Tai and others 2007). Microglial activation can be exacerbated by systemic LPS in a mouse model of HD, having no impact on the neurological symptoms (Franciosi and others 2012). A detrimental contribution of microglia in HD has been suggested, through complement-mediated neuronal damage, although supporting mechanistic evidence is limited (Singhrao and others 1999). Other in vitro studies have evidenced microglial proliferation and pro-inflammatory activation in HD, suggesting a reparative role in the removal of dysfunctional neurites at early and middle stages of the pathology (Kraft and others 2012). The current evidence supports the idea that microglial cells are activated during HD, but the question of whether the innate immune response is a bystander consequence or whether they have a direct effect on the disease progression is still a matter of debate and needs further research (Moller 2010). Interestingly, recent reports highlight the direct effect of mutant Huntingtin on the activation of microglia, suggesting a cell-autonomous regulation of the innate immune response in HD (Crotti and others 2014). The impact of systemic inflammatory events is clear during the progression of HD, as peripheral myeloid cells have been shown to produce altered levels of inflammatory cytokines (Trager and others 2014; Trager and Tabrizi 2013).

In summary, the study of the inflammatory response of microglial cells during brain pathology needs to keep on focusing the interest of scientific community in the future. The use of cell- and expression-profiling techniques in animal models of disease and a more detailed and advanced use of postmortem human samples at earlier stages of disease will help understand the key inflammatory pathways defining the microglial function in brain disease and to develop promising immunomodulatory therapeutic strategies.

## Concluding Remarks

The study of the functional states of microglia, and their effects on the physiology of the brain, in health and disease, are only starting to be understood. The successful application of technical and experimental innovation to the study of microglia has unveiled functions previously undefined, opening new avenues of research. To better understand, and define, the roles of microglia in the brain, we propose the study of their functions: surveillant microglia, proliferating microglia, pruning/neuromodulatory microglia, phagocytic microglia, and inflammatory microglia, and then the molecular profiles that are associated with these functions. Following this approach we would understand what microglia “are doing” rather than what they express, which will significantly overlap in many of these states, providing more precise information about their roles in health and disease. With a more or better definition of new functions, the future of the study of microglia cells is a promising and exciting arena that will determine how we understand brain function in health and disease.

## References

[bibr1-1073858414530512] Ajami B Bennett JL Krieger C McNagny KM Rossi FM 2011 Infiltrating monocytes trigger EAE progression, but do not contribute to the resident microglia pool. Nat Neurosci 14(9):1142–9. 21804537 10.1038/nn.2887

[bibr2-1073858414530512] Ajami B Bennett JL Krieger C Tetzlaff W Rossi FM 2007 Local self-renewal can sustain CNS microglia maintenance and function throughout adult life. Nat Neurosci 10(12):1538–43. 18026097 10.1038/nn2014

[bibr3-1073858414530512] Akiyama H Barger S Barnum S Bradt B Bauer J Cole GM, and others. 2000 Inflammation and Alzheimer’s disease. Neurobiol Aging 21(3):383–421. 10858586 10.1016/s0197-4580(00)00124-x PMC3887148

[bibr4-1073858414530512] Akiyama H Nishimura T Kondo H Ikeda K Hayashi Y McGeer PL 1994 Expression of the receptor for macrophage colony stimulating factor by brain microglia and its upregulation in brains of patients with Alzheimer’s disease and amyotrophic lateral sclerosis. Brain Res 639(1):171–4. 7514086 10.1016/0006-8993(94)91779-5

[bibr5-1073858414530512] Alliot F Godin I Pessac B. 1999 Microglia derive from progenitors, originating from the yolk sac, and which proliferate in the brain. Brain Res Dev Brain Res 117(2):145–52. 10.1016/s0165-3806(99)00113-3 10567732

[bibr6-1073858414530512] Audet JN Gowing G Paradis R Soucy G Julien JP 2012 Ablation of proliferating cells in the CNS exacerbates motor neuron disease caused by mutant superoxide dismutase. PLoS One 7(4):e34932. 22523565 10.1371/journal.pone.0034932 PMC3327706

[bibr7-1073858414530512] Bechade C Cantaut-Belarif Y Bessis A. 2013 Microglial control of neuronal activity. Front Cell Neurosci 7:32. 23543873 10.3389/fncel.2013.00032 PMC3610058

[bibr8-1073858414530512] Beers DR Henkel JS Xiao Q Zhao W Wang J Yen AA, and others. 2006 Wild-type microglia extend survival in PU.1 knockout mice with familial amyotrophic lateral sclerosis. Proc Natl Acad Sci U S A 103(43):16021–6. 17043238 10.1073/pnas.0607423103 PMC1613228

[bibr9-1073858414530512] Bhaskar K Konerth M Kokiko-Cochran ON Cardona A Ransohoff RM Lamb BT 2010 Regulation of tau pathology by the microglial fractalkine receptor. Neuron 68(1):19–31. 20920788 10.1016/j.neuron.2010.08.023 PMC2950825

[bibr10-1073858414530512] Billadeau DD Leibson PJ 2002 ITAMs versus ITIMs: striking a balance during cell regulation. J Clin Invest 109(2):161–8. 11805126 10.1172/JCI14843 PMC150845

[bibr11-1073858414530512] Blalock EM Geddes JW Chen KC Porter NM Markesbery WR Landfield PW 2004 Incipient Alzheimer’s disease: microarray correlation analyses reveal major transcriptional and tumor suppressor responses. Proc Natl Acad Sci U S A 101(7):2173–8. 14769913 10.1073/pnas.0308512100 PMC357071

[bibr12-1073858414530512] Blinzinger K Kreutzberg G. 1968 Displacement of synaptic terminals from regenerating motoneurons by microglial cells. Z Zellforsch Mikrosk Anat 85(2):145–57. 5706753 10.1007/BF00325030

[bibr13-1073858414530512] Boche D Perry VH Nicoll JA 2013 Review: activation patterns of microglia and their identification in the human brain. Neuropathol Appl Neurobiol 39(1):3–18. 23252647 10.1111/nan.12011

[bibr14-1073858414530512] Bolmont T Haiss F Eicke D Radde R Mathis CA Klunk WE, and others. 2008 Dynamics of the microglial/amyloid interaction indicate a role in plaque maintenance. J Neurosci 28(16):4283–92. 18417708 10.1523/JNEUROSCI.4814-07.2008 PMC3844768

[bibr15-1073858414530512] Brown GC Neher JJ 2012 Eaten alive! Cell death by primary phagocytosis: “phagoptosis.” Trends Biochem Sci 37(8):325–32. 22682109 10.1016/j.tibs.2012.05.002

[bibr16-1073858414530512] Butovsky O Jedrychowski MP Moore CS Cialic R Lanser AJ Gabriely G, and others. 2014 Identification of a unique TGF-beta-dependent molecular and functional signature in microglia. Nat Neurosci 17(1):131–43. 24316888 10.1038/nn.3599 PMC4066672

[bibr17-1073858414530512] Chan A Hummel V Weilbach FX Kieseier BC Gold R. 2006 Phagocytosis of apoptotic inflammatory cells downregulates microglial chemoattractive function and migration of encephalitogenic T cells. J Neurosci Res 84(6):1217–24. 16941488 10.1002/jnr.21029

[bibr18-1073858414530512] Chan WY Kohsaka S Rezaie P. 2007 The origin and cell lineage of microglia: new concepts. Brain Res Rev 53(2):344–54. 17188751 10.1016/j.brainresrev.2006.11.002

[bibr19-1073858414530512] Chung WS Clarke LE Wang GX Stafford BK Sher A Chakraborty C, and others. 2013 Astrocytes mediate synapse elimination through MEGF10 and MERTK pathways. Nature 504(7480):394–400. 24270812 10.1038/nature12776 PMC3969024

[bibr20-1073858414530512] Colton CA Mott RT Sharpe H Xu Q Van Nostrand WE Vitek MP 2006 Expression profiles for macrophage alternative activation genes in AD and in mouse models of AD. J Neuroinflammation 3:27. 17005052 10.1186/1742-2094-3-27 PMC1609108

[bibr21-1073858414530512] Cribbs DH Berchtold NC Perreau V Coleman PD Rogers J Tenner AJ, and others. 2012 Extensive innate immune gene activation accompanies brain aging, increasing vulnerability to cognitive decline and neurodegeneration: a microarray study. J Neuroinflammation 9:179. 10.1186/1742-2094-9-179 PMC3419089 22824372

[bibr22-1073858414530512] Crotti A Benner C Kerman BE Gosselin D Lagier-Tourenne C Zuccato C, and others. 2014 Mutant Huntingtin promotes autonomous microglia activation via myeloid lineage-determining factors. Nat Neurosci. 3 2. [Epub ahead of print] 10.1038/nn.3668 PMC4113004 24584051

[bibr23-1073858414530512] Crutcher KA Gendelman HE Kipnis J Perez-Polo JR Perry VH Popovich PG, and others. 2006 Debate: “Is increasing neuroinflammation beneficial for neural repair?” J Neuroimmune Pharmacol 1(3):195–211. 18040798 10.1007/s11481-006-9021-7

[bibr24-1073858414530512] Dalmau I Finsen B Zimmer J Gonzalez B Castellano B. 1998 Development of microglia in the postnatal rat hippocampus. Hippocampus 8(5):458–74. 9825958 10.1002/(SICI)1098-1063(1998)8:5<458::AID-HIPO6>3.0.CO;2-N

[bibr25-1073858414530512] Daneman R Zhou L Kebede AA Barres BA 2010 Pericytes are required for blood-brain barrier integrity during embryogenesis. Nature 468(7323):562–6. 20944625 10.1038/nature09513 PMC3241506

[bibr26-1073858414530512] Davalos D Grutzendler J Yang G Kim JV Zuo Y Jung S, and others. 2005 ATP mediates rapid microglial response to local brain injury in vivo. Nat Neurosci 8(6):752–8. 15895084 10.1038/nn1472

[bibr27-1073858414530512] Dawson TM Ko HS Dawson VL 2010 Genetic animal models of Parkinson’s disease. Neuron 66(5):646–61. 20547124 10.1016/j.neuron.2010.04.034 PMC2917798

[bibr28-1073858414530512] del Rio Hortega P 1932 Microglia. In: Penfield W, editor. Cytology and cellular pathology of the nervous system. New York, NY: Hoeber p 481–534.

[bibr29-1073858414530512] del Rio Hortega P Penfield W 1927 Cerebral cicatrix. The reaction of neuroglia and microglia to brain wounds. Bull Johns Hopkins Hosp 41:278–82.

[bibr30-1073858414530512] Del Rio-Hortega P 1920 La microglia y su transformacion en células en bastoncito y cuerpos granulo-adiposos. Trabajos del laboratorio de investigaciones biologicas 18(37).

[bibr31-1073858414530512] Dickson DW Lee SC Mattiace LA Yen SH Brosnan C. 1993 Microglia and cytokines in neurological disease, with special reference to AIDS and Alzheimer’s disease. Glia 7(1):75–83. 8423065 10.1002/glia.440070113

[bibr32-1073858414530512] Ding Z Mathur V Ho PP James ML Lucin KM Hoehne A, and others. 2014 Antiviral drug ganciclovir is a potent inhibitor of microglial proliferation and neuroinflammation. J Exp Med 211(2):189–98. 24493798 10.1084/jem.20120696 PMC3920559

[bibr33-1073858414530512] Edison P Archer HA Gerhard A Hinz R Pavese N Turkheimer FE, and others. 2008 Microglia, amyloid, and cognition in Alzheimer’s disease: an [11C](R)PK11195-PET and [11C]PIB-PET study. Neurobiol Dis 32(3):412–9. 18786637 10.1016/j.nbd.2008.08.001

[bibr34-1073858414530512] Esiri MM al Izzi MS Reading MC 1991 Macrophages, microglial cells, and HLA-DR antigens in fetal and infant brain. J Clin Pathol 44(2):102–6. 1864982 10.1136/jcp.44.2.102 PMC496969

[bibr35-1073858414530512] Fernandez-Botran R Ahmed Z Crespo FA Gatenbee C Gonzalez J Dickson DW, and others. 2011 Cytokine expression and microglial activation in progressive supranuclear palsy. Parkinsonism Relat Disord. 17:683–8. 21741294 10.1016/j.parkreldis.2011.06.007 PMC3196843

[bibr36-1073858414530512] Fiske BK Brunjes PC 2000 Microglial activation in the developing rat olfactory bulb. Neuroscience 96(4):807–15. 10727798 10.1016/s0306-4522(99)00601-6

[bibr37-1073858414530512] Fitzner D Schnaars M van Rossum D Krishnamoorthy G Dibaj P Bakhti M, and others. 2011 Selective transfer of exosomes from oligodendrocytes to microglia by macropinocytosis. J Cell Sci 124(Pt 3):447–58. 21242314 10.1242/jcs.074088

[bibr38-1073858414530512] Fontainhas AM Wang M Liang KJ Chen S Mettu P Damani M, and others. 2011 Microglial morphology and dynamic behavior is regulated by ionotropic glutamatergic and GABAergic neurotransmission. PLoS One 6(1):e15973. 21283568 10.1371/journal.pone.0015973 PMC3026789

[bibr39-1073858414530512] Franciosi S Ryu JK Shim Y Hill A Connolly C Hayden MR, and others. 2012 Age-dependent neurovascular abnormalities and altered microglial morphology in the YAC128 mouse model of Huntington disease. Neurobiol Dis 45(1):438–49. 21946335 10.1016/j.nbd.2011.09.003

[bibr40-1073858414530512] Frank S Burbach GJ Bonin M Walter M Streit W Bechmann I, and others. 2008 TREM2 is upregulated in amyloid plaque-associated microglia in aged APP23 transgenic mice. Glia 56(13):1438–47. 18551625 10.1002/glia.20710

[bibr41-1073858414530512] Frautschy SA Yang F Irrizarry M Hyman B Saido TC Hsiao K, and others. 1998 Microglial response to amyloid plaques in APPsw transgenic mice. Am J Pathol 152(1):307–17. 9422548 PMC1858113

[bibr42-1073858414530512] Fricker M Neher JJ Zhao JW Thery C Tolkovsky AM Brown GC 2012 MFG-E8 mediates primary phagocytosis of viable neurons during neuroinflammation. J Neurosci 32(8):2657–66. 22357850 10.1523/JNEUROSCI.4837-11.2012 PMC3312099

[bibr43-1073858414530512] Gerhard A Pavese N Hotton G Turkheimer F Es M Hammers A, and others. 2006 In vivo imaging of microglial activation with [11C](R)-PK11195 PET in idiopathic Parkinson’s disease. Neurobiol Dis 21(2):404–12. 16182554 10.1016/j.nbd.2005.08.002

[bibr44-1073858414530512] Ginhoux F Greter M Leboeuf M Nandi S See P Gokhan S, and others. 2010 Fate mapping analysis reveals that adult microglia derive from primitive macrophages. Science 330(6005):841–5. 20966214 10.1126/science.1194637 PMC3719181

[bibr45-1073858414530512] Ginhoux F Lim S Hoeffel G Low D Huber T. 2013 Origin and differentiation of microglia. Front Cell Neurosci 7:45. 23616747 10.3389/fncel.2013.00045 PMC3627983

[bibr46-1073858414530512] Gomez-Nicola D Fransen NL Suzzi S Perry VH 2013 Regulation of microglial proliferation during chronic neurodegeneration. J Neurosci 33(6):2481–93. 23392676 10.1523/JNEUROSCI.4440-12.2013 PMC6619184

[bibr47-1073858414530512] Gomez-Nicola D Schetters STT Perry VH 2014 Differential role of CCR2 in the dynamics of microglia and perivascular macrophages during prion disease. Glia. 3 19. 10.1002/glia.22660. [Epub ahead of print]. PMC4324129 24648328

[bibr48-1073858414530512] Gordon S 2003 Alternative activation of macrophages. Nat Rev Immunol 3(1):23–35. 12511873 10.1038/nri978

[bibr49-1073858414530512] Gowing G Lalancette-Hebert M Audet JN Dequen F Julien JP 2009 Macrophage colony stimulating factor (M-CSF) exacerbates ALS disease in a mouse model through altered responses of microglia expressing mutant superoxide dismutase. Exp Neurol 220(2):267–75. 19733170 10.1016/j.expneurol.2009.08.021

[bibr50-1073858414530512] Gowing G Philips T Van Wijmeersch B Audet JN Dewil M Van Den Bosch L, and others. 2008 Ablation of proliferating microglia does not affect motor neuron degeneration in amyotrophic lateral sclerosis caused by mutant superoxide dismutase. J Neurosci 28(41):10234–44. 18842883 10.1523/JNEUROSCI.3494-08.2008 PMC6671032

[bibr51-1073858414530512] Grathwohl SA Kalin RE Bolmont T Prokop S Winkelmann G Kaeser SA, and others. 2009 Formation and maintenance of Alzheimer’s disease beta-amyloid plaques in the absence of microglia. Nat Neurosci 12(11):1361–3. 19838177 10.1038/nn.2432 PMC4721582

[bibr52-1073858414530512] Griffin WS Sheng JG Roberts GW Mrak RE 1995 Interleukin-1 expression in different plaque types in Alzheimer’s disease: significance in plaque evolution. J Neuropathol Exp Neurol 54(2):276–81. 7876895 10.1097/00005072-199503000-00014

[bibr53-1073858414530512] Griffin WS Stanley LC Ling C White L MacLeod V Perrot LJ, and others. 1989 Brain interleukin 1 and S-100 immunoreactivity are elevated in Down syndrome and Alzheimer disease. Proc Natl Acad Sci U S A 86(19):7611–5. 2529544 10.1073/pnas.86.19.7611 PMC298116

[bibr54-1073858414530512] Guerreiro R Wojtas A Bras J Carrasquillo M Rogaeva E Majounie E, and others. 2013 TREM2 variants in Alzheimer’s disease. N Engl J Med 368(2):117–27. 23150934 10.1056/NEJMoa1211851 PMC3631573

[bibr55-1073858414530512] Hanisch UK Kettenmann H. 2007 Microglia: active sensor and versatile effector cells in the normal and pathologic brain. Nat Neurosci 10(11):1387–94. 17965659 10.1038/nn1997

[bibr56-1073858414530512] Hefendehl JK Neher JJ Suhs RB Kohsaka S Skodras A Jucker M. 2014 Homeostatic and injury-induced microglia behavior in the aging brain. Aging Cell 13(1):60–9. 23953759 10.1111/acel.12149 PMC4326865

[bibr57-1073858414530512] Heneka MT O’Banion MK 2007 Inflammatory processes in Alzheimer’s disease. J Neuroimmunol 184(1–2):69–91. 17222916 10.1016/j.jneuroim.2006.11.017

[bibr58-1073858414530512] Herber DL Roth LM Wilson D Wilson N Mason JE Morgan D, and others. 2004 Time-dependent reduction in Abeta levels after intracranial LPS administration in APP transgenic mice. Exp Neurol 190(1):245–53. 15473997 10.1016/j.expneurol.2004.07.007

[bibr59-1073858414530512] Hickman SE Kingery ND Ohsumi TK Borowsky ML Wang LC Means TK, and others. 2013 The microglial sensome revealed by direct RNA sequencing. Nat Neurosci 16(12):1896–905. 24162652 10.1038/nn.3554 PMC3840123

[bibr60-1073858414530512] Hines DJ Hines RM Mulligan SJ Macvicar BA 2009 Microglia processes block the spread of damage in the brain and require functional chloride channels. Glia 57(15):1610–8. 19382211 10.1002/glia.20874

[bibr61-1073858414530512] Holmes C Cunningham C Zotova E Culliford D Perry VH 2011 Proinflammatory cytokines, sickness behavior, and Alzheimer disease. Neurology 77(3):212–8. 21753171 10.1212/WNL.0b013e318225ae07 PMC3136056

[bibr62-1073858414530512] Holmes C Cunningham C Zotova E Woolford J Dean C Kerr S, and others. 2009 Systemic inflammation and disease progression in Alzheimer disease. Neurology 73(10):768–74. 19738171 10.1212/WNL.0b013e3181b6bb95 PMC2848584

[bibr63-1073858414530512] Hoozemans JJ Veerhuis R Rozemuller JM Eikelenboom P. 2011 Soothing the inflamed brain: effect of non-steroidal anti-inflammatory drugs on Alzheimer’s disease pathology. CNS Neurol Disord Drug Targets 10(1):57–67. 10.2174/187152711794488665 21143138

[bibr64-1073858414530512] Hosmane S Tegenge MA Rajbhandari L Uapinyoying P Kumar NG Thakor N, and others. 2012 Toll/interleukin-1 receptor domain-containing adapter inducing interferon-beta mediates microglial phagocytosis of degenerating axons. J Neurosci 32(22):7745–57. 22649252 10.1523/JNEUROSCI.0203-12.2012 PMC3398425

[bibr65-1073858414530512] Hua JY Smith SJ 2004 Neural activity and the dynamics of central nervous system development. Nat Neurosci 7(4):327–32. 15048120 10.1038/nn1218

[bibr66-1073858414530512] Hughes MM Field RH Perry VH Murray CL Cunningham C. 2010 Microglia in the degenerating brain are capable of phagocytosis of beads and of apoptotic cells, but do not efficiently remove PrPSc, even upon LPS stimulation. Glia 58(16):2017–30. 20878768 10.1002/glia.21070 PMC3498730

[bibr67-1073858414530512] Hunot S Boissiere F Faucheux B Brugg B Mouatt-Prigent A Agid Y, and others. 1996 Nitric oxide synthase and neuronal vulnerability in Parkinson’s disease. Neuroscience 72(2):355–63. 8737406 10.1016/0306-4522(95)00578-1

[bibr68-1073858414530512] Hutchins KD Dickson DW Rashbaum WK Lyman WD 1990 Localization of morphologically distinct microglial populations in the developing human fetal brain: implications for ontogeny. Brain Res Dev Brain Res 55(1):95–102. 10.1016/0165-3806(90)90109-c 2208643

[bibr69-1073858414530512] Jonsson T Stefansson H Steinberg S Jonsdottir I Jonsson PV Snaedal J, and others. 2013 Variant of TREM2 associated with the risk of Alzheimer’s disease. N Engl J Med 368(2):107–16. 23150908 10.1056/NEJMoa1211103 PMC3677583

[bibr70-1073858414530512] Jucker M 2010 The benefits and limitations of animal models for translational research in neurodegenerative diseases. Nat Med 16(11):1210–4. 21052075 10.1038/nm.2224

[bibr71-1073858414530512] Kamphuis W Orre M Kooijman L Dahmen M Hol EM 2012 Differential cell proliferation in the cortex of the APPswePS1dE9 Alzheimer’s disease mouse model. Glia 60(4):615–29. 22262260 10.1002/glia.22295

[bibr72-1073858414530512] Katz LC Shatz CJ 1996 Synaptic activity and the construction of cortical circuits. Science 274(5290):1133–8. 8895456 10.1126/science.274.5290.1133

[bibr73-1073858414530512] Kempermann G Jessberger S Steiner B Kronenberg G. 2004 Milestones of neuronal development in the adult hippocampus. Trends Neurosci 27(8):447–52. 15271491 10.1016/j.tins.2004.05.013

[bibr74-1073858414530512] Kettenmann H Hanisch UK Noda M Verkhratsky A. 2011 Physiology of microglia. Physiol Rev 91(2):461–553. 21527731 10.1152/physrev.00011.2010

[bibr75-1073858414530512] Kettenmann H Kirchhoff F Verkhratsky A. 2013 Microglia: new roles for the synaptic stripper. Neuron 77(1):10–8. 23312512 10.1016/j.neuron.2012.12.023

[bibr76-1073858414530512] Kierdorf K Erny D Goldmann T Sander V Schulz C Perdiguero EG, and others. 2013 Microglia emerge from erythromyeloid precursors via Pu.1- and Irf8-dependent pathways. Nat Neurosci 16(3):273–80. 23334579 10.1038/nn.3318

[bibr77-1073858414530512] Kraft AD Kaltenbach LS Lo DC Harry GJ 2012 Activated microglia proliferate at neurites of mutant huntingtin-expressing neurons. Neurobiol Aging 33(3):621 e17–e33. 21482444 10.1016/j.neurobiolaging.2011.02.015 PMC3135676

[bibr78-1073858414530512] Kreutzberg GW 1996 Microglia: a sensor for pathological events in the CNS. Trends Neurosci 19(8):312–8. 8843599 10.1016/0166-2236(96)10049-7

[bibr79-1073858414530512] LaFerla FM Oddo S. 2005 Alzheimer’s disease: Abeta, tau and synaptic dysfunction. Trends Mol Med 11(4):170–6. 15823755 10.1016/j.molmed.2005.02.009

[bibr80-1073858414530512] Lambert JC Heath S Even G Campion D Sleegers K Hiltunen M, and others. 2009 Genome-wide association study identifies variants at CLU and CR1 associated with Alzheimer’s disease. Nat Genet 41(10):1094–9. 19734903 10.1038/ng.439

[bibr81-1073858414530512] Lawson LJ Perry VH Dri P Gordon S. 1990 Heterogeneity in the distribution and morphology of microglia in the normal adult mouse brain. Neuroscience 39(1):151–70. 2089275 10.1016/0306-4522(90)90229-w

[bibr82-1073858414530512] Lawson LJ Perry VH Gordon S. 1992 Turnover of resident microglia in the normal adult mouse brain. Neuroscience 48(2):405–15. 1603325 10.1016/0306-4522(92)90500-2

[bibr83-1073858414530512] Li T Pang S Yu Y Wu X Guo J Zhang S. 2013 Proliferation of parenchymal microglia is the main source of microgliosis after ischaemic stroke. Brain 136(Pt 12):3578–88. 24154617 10.1093/brain/awt287

[bibr84-1073858414530512] Liang KJ Lee JE Wang YD Ma W Fontainhas AM Fariss RN, and others. 2009 Regulation of dynamic behavior of retinal microglia by CX3CR1 signaling. Invest Ophthalmol Vis Sci 50(9):4444–51. 19443728 10.1167/iovs.08-3357 PMC2749316

[bibr85-1073858414530512] Ling EA Penney D Leblond CP 1980 Use of carbon labeling to demonstrate the role of blood monocytes as precursors of the “ameboid cells” present in the corpus callosum of postnatal rats. J Comp Neurol 193(3):631–57. 6969268 10.1002/cne.901930304

[bibr86-1073858414530512] Maezawa I Jin LW 2010 Rett syndrome microglia damage dendrites and synapses by the elevated release of glutamate. J Neurosci 30(15):5346–56. 20392956 10.1523/JNEUROSCI.5966-09.2010 PMC5533099

[bibr87-1073858414530512] Magnus T Chan A Grauer O Toyka KV Gold R. 2001 Microglial phagocytosis of apoptotic inflammatory T cells leads to down-regulation of microglial immune activation. J Immunol 167(9):5004–10. 11673508 10.4049/jimmunol.167.9.5004

[bibr88-1073858414530512] McGeer PL Akiyama H Itagaki S McGeer EG 1989 Activation of the classical complement pathway in brain tissue of Alzheimer patients. Neurosci Lett 107(1–3):341–6. 2559373 10.1016/0304-3940(89)90843-4

[bibr89-1073858414530512] McGeer PL Itagaki S Boyes BE McGeer EG 1988 Reactive microglia are positive for HLA-DR in the substantia nigra of Parkinson’s and Alzheimer’s disease brains. Neurology 38(8):1285–91. 3399080 10.1212/wnl.38.8.1285

[bibr90-1073858414530512] McGeer PL McGeer EG 2002 The possible role of complement activation in Alzheimer disease. Trends Mol Med 8(11):519–23. 12421685 10.1016/s1471-4914(02)02422-x

[bibr91-1073858414530512] McGeer PL Schwab C Parent A Doudet D. 2003 Presence of reactive microglia in monkey substantia nigra years after 1-methyl-4-phenyl-1,2,3,6-tetrahydropyridine administration. Ann Neurol 54(5):599–604. 14595649 10.1002/ana.10728

[bibr92-1073858414530512] Mildner A Schlevogt B Kierdorf K Bottcher C Erny D Kummer MP, and others. 2011 Distinct and non-redundant roles of microglia and myeloid subsets in mouse models of Alzheimer’s disease. J Neurosci 31(31):11159–71. 21813677 10.1523/JNEUROSCI.6209-10.2011 PMC6623351

[bibr93-1073858414530512] Ming GL Song H. 2011 Adult neurogenesis in the mammalian brain: significant answers and significant questions. Neuron 70(4):687–702. 10.1016/j.neuron.2011.05.001 PMC3106107 21609825

[bibr94-1073858414530512] Mittelbronn M Dietz K Schluesener HJ Meyermann R. 2001 Local distribution of microglia in the normal adult human central nervous system differs by up to one order of magnitude. Acta Neuropathol 101(3):249–55. 11307625 10.1007/s004010000284

[bibr95-1073858414530512] Mizutani M Pino PA Saederup N Charo IF Ransohoff RM Cardona AE 2012 The fractalkine receptor but not CCR2 is present on microglia from embryonic development throughout adulthood. J Immunol 188(1):29–36. 22079990 10.4049/jimmunol.1100421 PMC3244524

[bibr96-1073858414530512] Mogi M Harada M Kondo T Riederer P Inagaki H Minami M, and others. 1994 Interleukin-1 beta, interleukin-6, epidermal growth factor and transforming growth factor-alpha are elevated in the brain from parkinsonian patients. Neurosci Lett 180(2):147–50. 7700568 10.1016/0304-3940(94)90508-8

[bibr97-1073858414530512] Moller T 2010 Neuroinflammation in Huntington’s disease. J Neural Transm 117(8):1001–8. 20535620 10.1007/s00702-010-0430-7

[bibr98-1073858414530512] Morris L Graham CF Gordon S. 1991 Macrophages in haemopoietic and other tissues of the developing mouse detected by the monoclonal antibody F4/80. Development 112(2):517–6. 1794320 10.1242/dev.112.2.517

[bibr99-1073858414530512] Neher JJ Emmrich JV Fricker M Mander PK Thery C Brown GC 2013 Phagocytosis executes delayed neuronal death after focal brain ischemia. Proc Natl Acad Sci U S A 110(43):E4098–107. 24101459 10.1073/pnas.1308679110 PMC3808587

[bibr100-1073858414530512] Neher JJ Neniskyte U Zhao JW Bal-Price A Tolkovsky AM Brown GC 2011 Inhibition of microglial phagocytosis is sufficient to prevent inflammatory neuronal death. J Immunol 186(8):4973–83. 21402900 10.4049/jimmunol.1003600

[bibr101-1073858414530512] Neumann H Misgeld T Matsumuro K Wekerle H. 1998 Neurotrophins inhibit major histocompatibility class II inducibility of microglia: involvement of the p75 neurotrophin receptor. Proc Natl Acad Sci U S A 95(10):5779–84. 10.1073/pnas.95.10.5779 PMC20456 9576961

[bibr102-1073858414530512] Nielsen HH Ladeby R Fenger C Toft-Hansen H Babcock AA Owens T, and others. 2009 Enhanced microglial clearance of myelin debris in T cell-infiltrated central nervous system. J Neuropathol Exp Neurol 68(8):845–56. 19606068 10.1097/NEN.0b013e3181ae0236

[bibr103-1073858414530512] Nimmerjahn A Kirchhoff F Helmchen F. 2005 Resting microglial cells are highly dynamic surveillants of brain parenchyma in vivo. Science 308(5726):1314–8. 15831717 10.1126/science.1110647

[bibr104-1073858414530512] Nissl F 1899 Ueber einige Beziehungen zwishcen Nerven zellerkrankungen und gliosen Erscheinnungen bei verschiedenen Psychosen. Arch Psychiat 32:1–21.

[bibr105-1073858414530512] Paolicelli RC Bolasco G Pagani F Maggi L Scianni M Panzanelli P, and others. 2011 Synaptic pruning by microglia is necessary for normal brain development. Science 333(6048):1456–8. 21778362 10.1126/science.1202529

[bibr106-1073858414530512] Pascual O Ben Achour S Rostaing P Triller A Bessis A. 2012 Microglia activation triggers astrocyte-mediated modulation of excitatory neurotransmission. Proc Natl Acad Sci U S A 109(4):E197–E205. 22167804 10.1073/pnas.1111098109 PMC3268269

[bibr107-1073858414530512] Pavese N Gerhard A Tai YF Ho AK Turkheimer F Barker RA, and others. 2006 Microglial activation correlates with severity in Huntington disease: a clinical and PET study. Neurology 66(11):1638–43. 10.1212/01.wnl.0000222734.56412.17 16769933

[bibr108-1073858414530512] Perry VH 2012 Innate inflammation in Parkinson’s disease. Cold Spring Harb Perspect Med 2(9):a009373. 22951445 10.1101/cshperspect.a009373 PMC3426823

[bibr109-1073858414530512] Perry VH Cunningham C Holmes C. 2007 Systemic infections and inflammation affect chronic neurodegeneration. Nat Rev Immunol 7(2):161–7. 17220915 10.1038/nri2015

[bibr110-1073858414530512] Perry VH Holmes C. 2014 Microglial priming in neurodegenerative disease. Nat Rev Neurol. 2014 3 18. 10.1038/nrneurol.2014.38. [Epub ahead of print]. 24638131

[bibr111-1073858414530512] Perry VH Hume DA Gordon S 1985 Immunohistochemical localization of macrophages and microglia in the adult and developing mouse brain. Neuroscience 15(2):313–26. 3895031 10.1016/0306-4522(85)90215-5

[bibr112-1073858414530512] Perry VH O’Connor V. 2010 The role of microglia in synaptic stripping and synaptic degeneration: a revised perspective. ASN Neuro 2(5):e00047. 20967131 10.1042/AN20100024 PMC2954441

[bibr113-1073858414530512] Perry VH Teeling J. 2013 Microglia and macrophages of the central nervous system: the contribution of microglia priming and systemic inflammation to chronic neurodegeneration. Semin Immunopathol 35(5):601–12. 23732506 10.1007/s00281-013-0382-8 PMC3742955

[bibr114-1073858414530512] Pocock JM Kettenmann H. 2007 Neurotransmitter receptors on microglia. Trends Neurosci 30(10):527–35. 17904651 10.1016/j.tins.2007.07.007

[bibr115-1073858414530512] Politis M Pavese N Tai YF Kiferle L Mason SL Brooks DJ, and others. 2011 Microglial activation in regions related to cognitive function predicts disease onset in Huntington’s disease: a multimodal imaging study. Hum Brain Mapp 32(2):258–70. 21229614 10.1002/hbm.21008 PMC6870088

[bibr116-1073858414530512] Popovich PG Longbrake EE 2008 Can the immune system be harnessed to repair the CNS? Nat Rev Neurosci 9(6):481–93. 18490917 10.1038/nrn2398

[bibr117-1073858414530512] Pott Godoy MC Tarelli R Ferrari CC Sarchi MI Pitossi FJ 2008 Central and systemic IL-1 exacerbates neurodegeneration and motor symptoms in a model of Parkinson’s disease. Brain 131(Pt 7):1880–94. 18504291 10.1093/brain/awn101 PMC2442423

[bibr118-1073858414530512] Prinz M Mildner A. 2011 Microglia in the CNS: immigrants from another world. Glia 59(2):177–87. 21125659 10.1002/glia.21104

[bibr119-1073858414530512] Ransohoff RM Perry VH 2009 Microglial physiology: unique stimuli, specialized responses. Annu Rev Immunol 27:119–45. 19302036 10.1146/annurev.immunol.021908.132528

[bibr120-1073858414530512] Rezaie P Male D. 1999 Colonisation of the developing human brain and spinal cord by microglia: a review. Microsc Res Tech 45(6):359–82. 10402264 10.1002/(SICI)1097-0029(19990615)45:6<359::AID-JEMT4>3.0.CO;2-D

[bibr121-1073858414530512] Robertson W 1900 A microscopic demonstration of the normal and pathological histology of mesoglia cells. J Ment Sci 46:733–52.

[bibr122-1073858414530512] Sapp E Kegel KB Aronin N Hashikawa T Uchiyama Y Tohyama K, and others. 2001 Early and progressive accumulation of reactive microglia in the Huntington disease brain. J Neuropathol Exp Neurol 60(2):161–72. 11273004 10.1093/jnen/60.2.161

[bibr123-1073858414530512] Schafer DP Lehrman EK Kautzman AG Koyama R Mardinly AR Yamasaki R, and others. 2012 Microglia sculpt postnatal neural circuits in an activity and complement-dependent manner. Neuron 74(4):691–705. 22632727 10.1016/j.neuron.2012.03.026 PMC3528177

[bibr124-1073858414530512] Schafer DP Lehrman EK Stevens B. 2013 The “quad-partite” synapse: microglia-synapse interactions in the developing and mature CNS. Glia 61(1):24–36. 22829357 10.1002/glia.22389 PMC4082974

[bibr125-1073858414530512] Schulz C Gomez Perdiguero E Chorro L Szabo-Rogers H Cagnard N Kierdorf K, and others. 2012 A lineage of myeloid cells independent of Myb and hematopoietic stem cells. Science 336(6077):86–90. 22442384 10.1126/science.1219179

[bibr126-1073858414530512] Shaftel SS Kyrkanides S Olschowka JA Miller JN Johnson RE O’Banion MK 2007 Sustained hippocampal IL-1 beta overexpression mediates chronic neuroinflammation and ameliorates Alzheimer plaque pathology. J Clin Invest 117(6):1595–604. 17549256 10.1172/JCI31450 PMC1878531

[bibr127-1073858414530512] Sierra A Abiega O Shahraz A Neumann H. 2013 Janus-faced microglia: beneficial and detrimental consequences of microglial phagocytosis. Front Cell Neurosci 7:6. 23386811 10.3389/fncel.2013.00006 PMC3558702

[bibr128-1073858414530512] Sierra A Encinas JM Deudero JJ Chancey JH Enikolopov G Overstreet-Wadiche LS, and others. 2010 Microglia shape adult hippocampal neurogenesis through apoptosis-coupled phagocytosis. Cell Stem Cell 7(4):483–95. 20887954 10.1016/j.stem.2010.08.014 PMC4008496

[bibr129-1073858414530512] Simard AR Rivest S. 2006 Neuroprotective properties of the innate immune system and bone marrow stem cells in Alzheimer’s disease. Mol Psychiatry 11(4):327–35. 16491130 10.1038/sj.mp.4001809

[bibr130-1073858414530512] Simard AR Soulet D Gowing G Julien JP Rivest S. 2006 Bone marrow-derived microglia play a critical role in restricting senile plaque formation in Alzheimer’s disease. Neuron 49(4):489–502. 16476660 10.1016/j.neuron.2006.01.022

[bibr131-1073858414530512] Singhrao SK Neal JW Morgan BP Gasque P. 1999 Increased complement biosynthesis by microglia and complement activation on neurons in Huntington’s disease. Exp Neurol 159(2):362–76. 10506508 10.1006/exnr.1999.7170

[bibr132-1073858414530512] Siskova Z Page A O’Connor V Perry VH 2009 Degenerating synaptic boutons in prion disease: microglia activation without synaptic stripping. Am J Pathol 175(4):1610–21. 19779137 10.2353/ajpath.2009.090372 PMC2751557

[bibr133-1073858414530512] Sogn CJ Puchades M Gundersen V. 2013 Rare contacts between synapses and microglial processes containing high levels of Iba1 and actin—a postembedding immunogold study in the healthy rat brain. Eur J Neurosci 38(1):2030–40. 23590220 10.1111/ejn.12213

[bibr134-1073858414530512] Sriram K Miller DB O’Callaghan JP 2006 Minocycline attenuates microglial activation but fails to mitigate striatal dopaminergic neurotoxicity: role of tumor necrosis factor-alpha. J Neurochem 96(3):706–18. 16405514 10.1111/j.1471-4159.2005.03566.x

[bibr135-1073858414530512] Stevens B Allen NJ Vazquez LE Howell GR Christopherson KS Nouri N, and others. 2007 The classical complement cascade mediates CNS synapse elimination. Cell 131(6):1164–78. 18083105 10.1016/j.cell.2007.10.036

[bibr136-1073858414530512] Su X Maguire-Zeiss KA Giuliano R Prifti L Venkatesh K Federoff HJ 2008 Synuclein activates microglia in a model of Parkinson’s disease. Neurobiol Aging 29(11):1690–701. 17537546 10.1016/j.neurobiolaging.2007.04.006 PMC2621109

[bibr137-1073858414530512] Sudduth TL Schmitt FA Nelson PT Wilcock DM 2013 Neuroinflammatory phenotype in early Alzheimer’s disease. Neurobiol Aging 34(4):1051–9. 23062700 10.1016/j.neurobiolaging.2012.09.012 PMC3579221

[bibr138-1073858414530512] Tai YF Pavese N Gerhard A Tabrizi SJ Barker RA Brooks DJ, and others. 2007 Microglial activation in presymptomatic Huntington’s disease gene carriers. Brain 130(Pt 7):1759–66. 17400599 10.1093/brain/awm044

[bibr139-1073858414530512] Tambuyzer BR Ponsaerts P Nouwen EJ 2009 Microglia: gatekeepers of central nervous system immunology. J Leukoc Biol 85(3):352–70. 19028958 10.1189/jlb.0608385

[bibr140-1073858414530512] Trager U Andre R Lahiri N Magnusson-Lind A Weiss A Grueninger S, and others. 2014 HTT-lowering reverses Huntington’s disease immune dysfunction caused by NFkappaB pathway dysregulation. Brain 137(Pt 3):819–33. 24459107 10.1093/brain/awt355 PMC3983408

[bibr141-1073858414530512] Trager U Tabrizi SJ 2013 Peripheral inflammation in neurodegeneration. J Mol Med (Berl) 91(6):673–81. 23546523 10.1007/s00109-013-1026-0

[bibr142-1073858414530512] Tremblay ME Lowery RL Majewska AK 2010 Microglial interactions with synapses are modulated by visual experience. PLoS Biol 8(11):e1000527. 21072242 10.1371/journal.pbio.1000527 PMC2970556

[bibr143-1073858414530512] Tremblay ME Zettel ML Ison JR Allen PD Majewska AK 2012 Effects of aging and sensory loss on glial cells in mouse visual and auditory cortices. Glia 60(4):541–58. 22223464 10.1002/glia.22287 PMC3276747

[bibr144-1073858414530512] Wake H Moorhouse AJ Jinno S Kohsaka S Nabekura J. 2009 Resting microglia directly monitor the functional state of synapses in vivo and determine the fate of ischemic terminals. J Neurosci 29(13):3974–80. 19339593 10.1523/JNEUROSCI.4363-08.2009 PMC6665392

[bibr145-1073858414530512] Walsh S Finn DP Dowd E. 2011 Time-course of nigrostriatal neurodegeneration and neuroinflammation in the 6-hydroxydopamine-induced axonal and terminal lesion models of Parkinson’s disease in the rat. Neuroscience 175:251–61. 21145947 10.1016/j.neuroscience.2010.12.005

[bibr146-1073858414530512] Wang CC Wu CH Shieh JY Wen CY Ling EA 1996 Immunohistochemical study of amoeboid microglial cells in fetal rat brain. J Anat 189(Pt 3):567–74. 8982832 PMC1167699

[bibr147-1073858414530512] Wang G Zhang Y Chen B Cheng J. 2003 Preliminary studies on Alzheimer’s disease using cDNA microarrays. Mech Ageing Dev 124(1):115–24. 12618014 10.1016/s0047-6374(02)00188-4

[bibr148-1073858414530512] Wei R Jonakait GM 1999 Neurotrophins and the anti-inflammatory agents interleukin-4 (IL-4), IL-10, IL-11 and transforming growth factor-beta1 (TGF-beta1) down-regulate T cell costimulatory molecules B7 and CD40 on cultured rat microglia. J Neuroimmunol 95(1–2):8–18. 10229111 10.1016/s0165-5728(98)00248-3

[bibr149-1073858414530512] Wu DC Jackson-Lewis V Vila M Tieu K Teismann P Vadseth C, and others. 2002 Blockade of microglial activation is neuroprotective in the 1-methyl-4-phenyl-1,2,3,6-tetrahydropyridine mouse model of Parkinson disease. J Neurosci 22(5):1763–71. 11880505 10.1523/JNEUROSCI.22-05-01763.2002 PMC6758858

[bibr150-1073858414530512] Yamada J Nakanishi H Jinno S. 2011 Differential involvement of perineuronal astrocytes and microglia in synaptic stripping after hypoglossal axotomy. Neuroscience 182:1–10. 21435379 10.1016/j.neuroscience.2011.03.030

[bibr151-1073858414530512] Zhan Y Paolicelli RC Sforazzini F Weinhard L Bolasco G Pagani F, and others. 2014 Deficient neuron-microglia signaling results in impaired functional brain connectivity and social behavior. Nat Neurosci 17(3):400–6. 24487234 10.1038/nn.3641

[bibr152-1073858414530512] Zhang S Wang XJ Tian LP Pan J Lu GQ Zhang YJ, and others. 2011 CD200-CD200R dysfunction exacerbates microglial activation and dopaminergic neurodegeneration in a rat model of Parkinson’s disease. J Neuroinflammation 8:154. 22053982 10.1186/1742-2094-8-154 PMC3226566

